# Research on the Algorithm Model for Measuring Ocean Waves Based on Satellite GPS Signals in China

**DOI:** 10.3390/s19030541

**Published:** 2019-01-28

**Authors:** Zhanhui Qi, Shaowu Li, Mingbing Li, Chaoqun Dang, Dongbo Sun, Dongliang Zhang, Ning Liu, Suoping Zhang

**Affiliations:** 1 School of Civil Engineering, Tianjin University, Yaguan Road No.135, Haihe Education Park, Jinnan District, Tianjin 300350, China; lishaowu@tju.edu.cn; 2 National Ocean Technology Center, Jieyuanxidao Road No.219, Nankai District, Tianjin 300112, China; limingbing@126.com (M.L.); dangchaoqun06@163.com (C.D.); dongbosun@163.com (D.S.); zhangdongliang@126.com (D.Z.); liun0130@126.com (N.L.); iot232@126.com (S.Z.)

**Keywords:** GPS, wave buoy, Doppler frequency shift, wave height, wave period, wave direction

## Abstract

In recent years, the GPS wave buoy has been developed for in situ wave monitoring based on satellite GPS signals. Many research works have been completed on the GPS-based wave measurement technology and great progress has been achieved. The basic principle of the GPS wave buoy is to calculate the movement velocity of the buoy using the Doppler frequency shift of satellite GPS signals, and then to calculate the wave parameters from the movement velocity according to ocean wave theory. The shortage of the GPS wave buoy is the occasional occurrence of some unusual values in the movement velocity. This is mainly due to the fact that the GPS antenna is occasionally covered by sea water and cannot normally receive high-quality satellite GPS signals. The traditional solution is to remove these unusual movement velocity values from the records, which requires furthering extend the acquisition time of satellite GPS signals to ensure there is a large enough quantity of effective movement velocity values. Based on the traditional GPS wave measurement technology, this paper presents the algorithmic flow and proposes two improvement measures. On the one hand, the neural network algorithm is used to correct the unusual movement velocity data so that extending the acquisition time of satellite GPS signals is not necessary and battery power is saved. On the other hand, the Gaussian low-pass filter is used to correct the raw directional wave spectrum, which can further eliminate the influence of noise spectrum energy and improve the measurement accuracy. The on-site sea test of the SBF7-1A GPS wave buoy, developed by the National Ocean Technology Center in China, and the gravity-acceleration-type DWR-MKIII Waverider buoy are highlighted in this article. The wave data acquired by the two buoys are analyzed and processed. It can be seen from the processed results that the ocean wave parameters from the two kinds of wave buoys, such as wave height, wave period, wave direction, wave frequency spectrum, and directional wave spectrum, are in good consistency, indicating that the SBF7-1A GPS wave buoy is comparable to the traditional gravity-acceleration-type wave buoy in terms of its accuracy. Therefore, the feasibility and validity of the two improvement measures proposed in this paper are confirmed.

## 1. Introduction

Ocean waves are a very important dynamic factor in the marine environment. Enormous amounts of casualties and economic property losses have been directly caused by ocean waves in marine disasters over the years because coastal areas are usually densely populated economic centers. Moreover, the stability of marine structures closely depends on the loads generated by waves. Waves are also the main driving force for sediment and contaminant transport. Therefore, it is a priority to obtain accurate in situ ocean wave data to improve the ability to prevent ocean wave disasters, provide reasonable stability for coastal structures, and guarantee the safety of people’s lives and property in coastal regions [1].

The wave buoy has the advantage of not being limited by water depth and field environment. Consequently, it has been widely used in the field observations of ocean waves. The wave buoy is designed with a spherical shape to ensure good wave-following performance in adapting to the circular motion of water-wave particles [2,3].

Over the years, the traditional gravity-acceleration-type wave buoy has been recognized as one of the most reliable instruments with reasonable accuracy in monitoring in situ wave parameters, including wave height, wave period, and wave direction. The device, mounted with an acceleration sensor, an electronic compass, a gyroscope, and other sensors, measures waves based on the principle of gravity acceleration. Currently, several brands of this type of wave buoy are available, such as the DWR-MKIII Waverider buoy developed by the DataWell BV company of the Netherlands [4], the Triaxys wave buoy with a combination of solar and battery power supply for longer performance produced by the Canadian AXYS Corporation [5], the SBY6-1 and the SBF7-1 wave buoys developed by the National Ocean Technology Center of China, the SBF3 wave buoy with two types of anchor connection for different environmental applications developed by the Institute of Oceanographic Instrumentation, Shandong Academy of Sciences (SDIOI) of China [6], and the SZF wave buoy developed by the Ocean University of China [7].

In recent years, a new wave measurement method has been invented that uses satellite GPS signals to measure ocean waves, namely, the GPS wave buoy. The measuring principle of the GPS wave buoy is to calculate the movement velocity of the buoy using the Doppler frequency shift of satellite GPS signals, and then to calculate wave parameters, such as wave height, wave period, wave direction, wave frequency spectrum, and directional wave spectrum from the movement velocity according to ocean wave theory. The GPS wave buoy, which is a simple, small-sized hardware device, only needs a GPS sensor without the mounting of any other auxiliary sensors. Since it measures waves from received satellite GPS signals, it is easy to install and maintain. Compared to the traditional gravity-acceleration-type wave buoy, the GPS wave buoy has the following characteristics. First, satellite GPS signals do not further increase measurement errors due to increases in usage time, so the GPS wave buoy does not need to be regularly calibrated. The gravity-acceleration-type wave buoy needs to be calibrated again after a period of use. The GPS wave buoy is also not affected by the on-site magnetic environment, while the gravity-acceleration-type wave buoy is because it has an electronic compass sensor. Finally, the GPS wave buoy is capable of measuring longer period waves relative to the gravity-acceleration-type wave buoy [4]. At present, many research institutes have carried out research work on GPS wave measurement technology and have made great progress. In particular, the DataWell BV company has developed various types of GPS wave buoys with different buoy diameters of 0.4 m (DWR-G4), 0.7 m (DWR-G7), and 0.9m (DWR-G9) [4]. At the same time, a number of on-site sea comparison tests have been conducted on the GPS wave buoy, which have achieved very good results [8,9,10,11,12,13,14].

In the use of GPS wave buoy, it is found that the GPS wave buoy often requires a longer data acquisition time of satellite GPS signals in comparison with the traditional gravity-acceleration-type wave buoy. This is mainly due to the fact that the GPS wave buoy directly removes some unusual movement velocity values in tradition, thereby extending the data acquisition time. The GPS antenna of the GPS wave buoy is occasionally covered by sea water because of its low height from the sea surface. Therefore, the GPS wave buoy cannot receive high-quality satellite GPS signals occasionally, and thus cannot resolve high-precision movement velocity data of the buoy, namely, unusual movement velocity data. In order to ensure the accuracy of measuring waves, the traditional data-preprocessing method is to directly remove these unusual movement velocity values. Therefore, it requires longer data-acquisition time to collect enough normal movement velocity values. The wave measurement effect using the traditional data-preprocessing method is very good, so this method is very worthy of recognition [9]. 

This paper proposes a new data-preprocessing method that is different from the traditional method. The method uses a neural network algorithm to correct unusual movement velocity data. Therefore, it does not need to additionally extend the satellite signals data-acquisition time of the GPS wave buoy. This can further save the energy of the GPS wave buoy and prolong the cycle of replacing the buoy battery. At the same time, another improvement proposed in this paper is to introduce a Gaussian low-pass filter in the processing of the raw directional wave spectrum, which further reduces the influence of the noise wave spectrum energy and improves the accuracy of wave measurement.

Based on the above two improvement measures proposed in this article, the National Ocean Technology Center in China has also carried out research work on GPS wave measurement technology and has made great progress [15,16], successfully developing the SBF7-1A GPS wave buoy. An on-site comparison test was carried out for the SBF7-1A GPS wave buoy and the gravity-acceleration-type DWR-MKIII Waverider buoy. The wave height, wave period, wave direction, wave frequency spectrum, and directional wave spectrum from the two buoys had fairly good consistency. The correlation coefficients reached 0.97, 0.98, and 0.89 in significant wave height, mean wave period, and wave direction, respectively. Thus, the effectiveness of the two improvements has been further verified.

After having obtained certifications from third-party verification agencies, e.g., the National Center Of Ocean Standards and Metrology, the National Institute of Metrology (NIM), and the National Quality Supervision and Inspection Center for Marine Instruments and Equipment, the SBF7-1A GPS wave buoy is presently used by many military and civilian ocean-observation sites, such as the navy, air force, the State Oceanic Administration, the National Offshore Maritime Comprehensive Testing Site, and the ocean engineering corporation, and has achieved very good results. At the same time, a number of drifting-type SBF7-1A GPS wave buoys have been used in the South China Sea, the Western Pacific, and the Indian Ocean. These buoys are still quite stable after experiencing many typhoon processes. The performances of the SBF7-1A GPS buoys have proven to be stable and reliable [17,18].

This paper is organized as follows. The theory and algorithm for measuring ocean waves by satellite GPS signals are described in Section 2 and Section 4. Section 3, Section 5 and Section 6 present on-site sea trials, data processing, and results analysis. The discussion and conclusion are presented in Section 7 and Section 8, respectively.

## 2. Materials and Methods

In China, the National Ocean Technology Center started researching this topic in 2009, and has gradually mastered the key core technologies and successfully developed the SBF7-1A GPS wave buoy.

**Figure 1:**
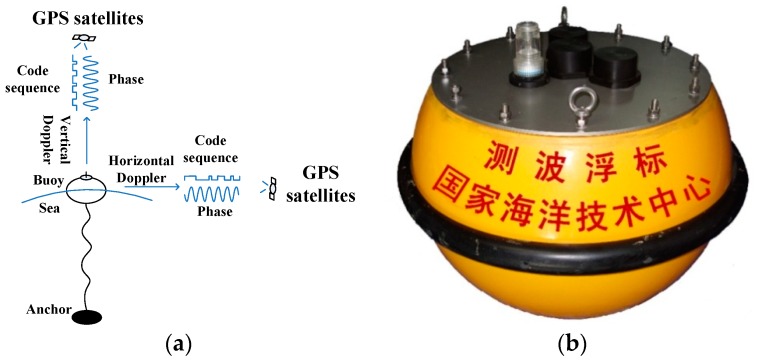
Measurement mechanism of the SBF7-1A GPS wave buoy developed by the National Ocean Technology Center in China: (**a**) schematic diagram; (**b**) physical picture.

The basic measurement principle of the SBF7-1A GPS wave buoy is shown in Figure 1. The wave buoy floats on the sea. The GPS sensor in the wave buoy receives satellite GPS signals from multiple satellites in multiple directions. Due to the relative motion between the wave buoy and the satellites, there is a frequency difference between the satellite GPS signals received by the GPS wave buoy and the satellite GPS signals sent by the GPS satellites, namely, the Doppler frequency shift. The magnitude of the Doppler frequency shift is inherently related to the movement velocity of the wave buoy. Based on this intrinsic relation, the buoy’s movement velocity can be calculated from the Doppler frequency shift. Then, wave parameters such as wave height, wave period, and wave direction can be obtained from the movement velocity of the buoy.

Therefore, using the Doppler frequency shift value to calculate movement velocity is very critical for the GPS wave buoy. After selective availability (SA) is cancelled, the measurement accuracy of the movement velocity using satellite GPS signals can reach the level of cm/s, even mm/s [19,20,21,22,23,24]. Velocity-measurement accuracy can meet wave-measurement requirements, which makes it possible to measure ocean waves with satellite GPS signals.

The basic calculation process is as follows. It is streamlined compared to the traditional measurement algorithm flow.

According to the principle of the Doppler frequency shift, the buoy’s movement velocity components $V_{1}\left(t\right),$ $V_{2}\left(t\right)$, and $V_{3}\left(t\right)$ are calculated from the satellite GPS signals. $V_{1}\left(t\right),$ $V_{2}\left(t\right)$, and $V_{3}\left(t\right)$ are the velocity components of the buoy’s movement in the east–west, north–south, and vertical direction, respectively.

The three movement-velocity data sequences $V_{1}\left(t\right),$ $V_{2}\left(t\right)$, and $V_{3}\left(t\right)$ are preprocessed with a neural network algorithm to remove unusual values.

The wave spectrum can be obtained from the cross-spectrum, which is the Fourier transform of the cross-correlation function. The cross-correlation function is defined as follows:

(1) $$R_{mn}\left(\tau \right)=\underset{T\to \infty }{\lim}\frac{1}{T}\int_{-T/2}^{T/2}V_{m}\left(t\right)V_{n}\left(t+\tau \right)dt,\;m,n=1,2,3\;$$

The cross-spectrum $S_{mn}\left(f\right)$ is defined as follows:

(2) $$S_{mn}\left(f\right)=\int_{-\infty }^{\infty }R_{mn}\left(\tau \right)e^{-2\pi if\tau }d\tau =C_{mn}\left(f\right)-iQ_{mn}\left(f\right),\;m,n=1,2,3$$

Therefore, six sets of cross-spectrum can be obtained from the combination of the three movement velocity components of the GPS wave buoy.

The raw directional wave spectrum $S^{\prime }\left(f,\theta \right)$ is obtained from the six sets of cross-spectrum calculated using Equation (3):

(3) $$S^{\prime }\left(f,\theta \right)=A_{0}\left(f\right)+A_{1}\left(f\right)\cos(\theta )+B_{1}\left(f\right)\sin(\theta )+A_{2}\left(f\right)\cos(2\theta )+B_{2}\left(f\right)\sin(2\theta )$$

where coefficients $A_{0}\left(f\right)$, $A_{1}\left(f\right)$, $B_{1}\left(f\right)$, $A_{2}\left(f\right)$, and $B_{2}\left(f\right)$ can be obtained from Equations (4)–(8).

(4) $$A_{0}\left(f\right)=\frac{C_{11}\left(f\right)}{\pi }$$

(5) $$A_{1}\left(f\right)=\frac{Q_{12}\left(f\right)}{\pi k}$$

(6) $$A_{2}\left(f\right)=\frac{C_{22}\left(f\right)-C_{33}\left(f\right)}{\pi k^{2}}$$

(7) $$B_{1}\left(f\right)=\frac{Q_{13}\left(f\right)}{\pi k}$$

(8) $$B_{2}\left(f\right)=\frac{2C_{23}\left(f\right)}{\pi k^{2}}$$

The raw directional wave spectrum $S^{\prime }\left(f,\theta \right)$ is smoothed by a Gaussian low-pass filter to obtain a new directional wave spectrum S(f,θ).

The wave frequency spectrum S(f) can be obtained by integrating the directional wave spectrum S(f,θ) with respect to direction angular *θ*.

From the wave frequency spectrum S(f), the wave height and wave period are obtained as follows:

(9) $$\mathrm{The}\;\mathrm{significant}\;\mathrm{wave}\;\mathrm{height}:\;H_{s}=4\sqrt{m_{0}}$$

(10) $$\mathrm{The}\;\mathrm{mean}\;\mathrm{wave}\;\mathrm{period}:\;\bar{T}=2\pi \sqrt{\frac{m_{0}}{m_{2}}}$$

where $m_{n}$ is the nth-order spectral momentum of the wave energy density spectrum.

(11) $$m_{n}=\int_{0}^{\infty }\omega^{n}S\left(\omega \right)d\omega$$

Directional wave spectrum S(f,θ) can be viewed as a wave frequency spectrum S(f) multiplied by the wave directional spreading function D(θ,f), as shown below:

(12) S(f,θ)=S(f)D(θ,f)

When directional wave spectrum S(f,θ) and wave frequency spectrum S(f) are obtained, wave directional spreading function D(θ,f) can be obtained according to Equation (12).

## 3. In Situ Comparison Test and Results without a Neural-Network Algorithm and a Gaussian Low-Pass Filter

**Figure 2:**
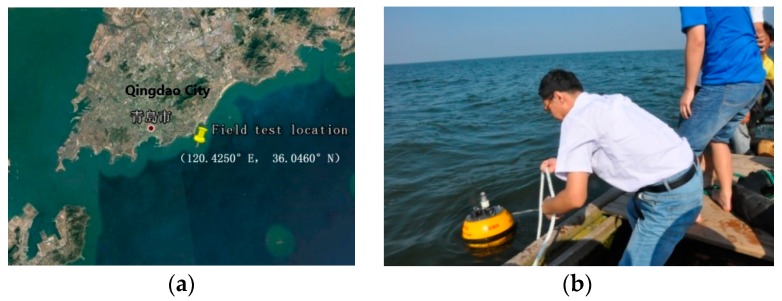
Field test between the SBF7-1A GPS wave buoy and the DWR-MKIII Waverider buoy: (**a**) field test location; (**b**) placing the buoy.

The SBF7-1A GPS wave buoy and the gravity-acceleration-type DWR-MKIII Waverider buoy were mounted in the field to simultaneously monitor wave data, as shown in Figure 2. The two wave buoys used the same mooring system, which was composed of a buoyancy ball, an elastic cable, a nylon rope, and a grip anchor. Data acquisition lasted from 19 June to 3 July 2013 for about 14 days, with an acquisition interval of 1 h. The distance between the two buoys was about 500 m and the water depth in the sea area was about 20 m.

The SBF7-1A GPS wave buoy and the DWR-MKIII Waverider buoy calculate the wave parameters from the self-moving state of the buoy on the sea surface. They all belong to the wave observation device of the buoy.

The calculation process of the DWR-MKIII Waverider buoy is as follows. Three sets of motion displacement of the buoy in the east-west, north-south, and vertical directions are obtained from integrating twice the three sets of motion acceleration captured by the DWR-MKIII Waverider buoy, respectively. The directional wave spectrum of the buoy is calculated from three sets of motion displacement, and the wave height, wave period, and wave direction are therefore calculated from the directional wave spectrum.

**Figure 3:**
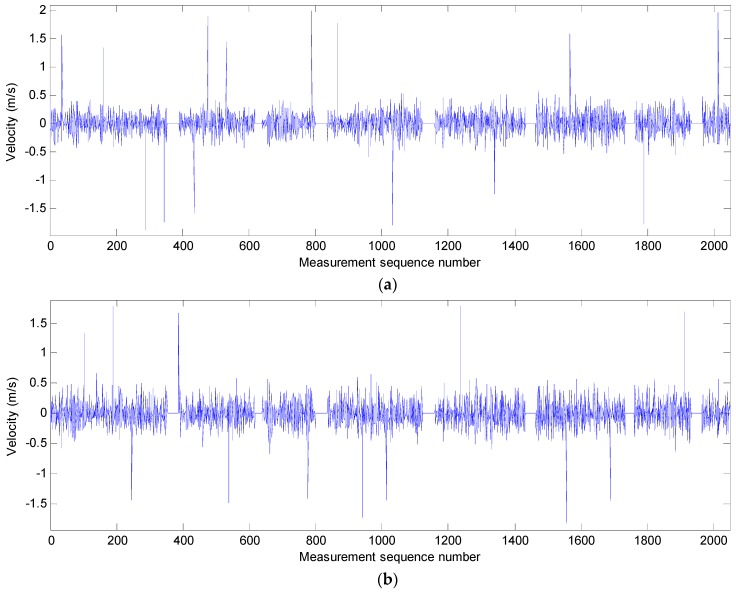

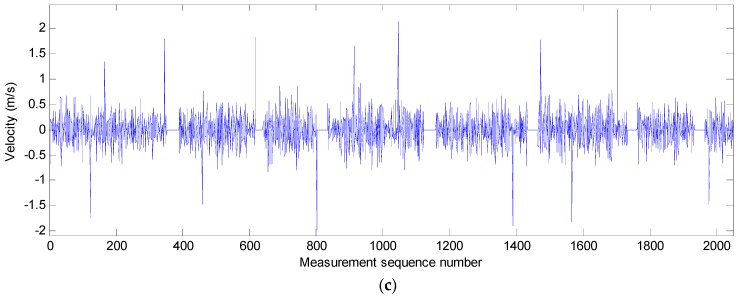
Movement velocity data sequences of the SBF7-1A GPS wave buoy in three directions before correction at 03:00 (UTC) on 2 July 2013: (**a**) movement velocity data sequence in the east–west direction; (**b**) movement velocity data sequence in the north–south direction; and (**c**) movement velocity data sequence in the vertical direction.

The three raw movement velocity data sequences of the SBF7-1A GPS wave buoy at 03:00 (UTC) on 2 July 2013 are shown in Figure 3. As can be seen from these data sequences, sudden and major increases of the velocity values occur at some points. Moreover, some zero value points also occurred at several data segments, which is believed to be caused by occasional hitting of the wave overtopping on the GPS antenna when the buoy moves at a resonance status under the complex action of the waves, currents, and the mooring lines. These rapid increases of the velocity data and null velocity data segments with a value of 0 all belong to the unusual data.

**Figure 4:**
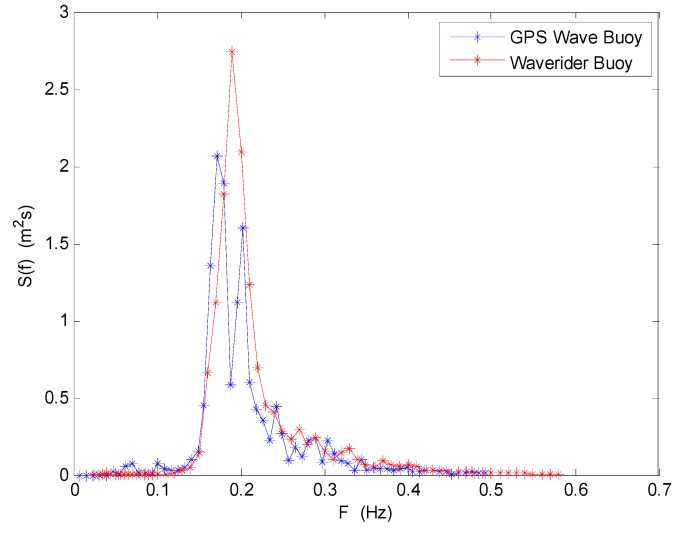
Comparison of wave frequency spectrum between the SBF7-1A GPS wave buoy and the DWR-MKIII Waverider buoy before correction at 03:00 (UTC) on 2 July 2013.

Without using a neural network algorithm and a Gaussian low-pass filter, the wave frequency spectrum and the directional wave spectrum are directly calculated from the three raw movement velocity data sequences. The wave frequency spectrum of the two wave buoys at 03:00 (UTC) on 2 July 2013 are shown in Figure 4. They do not have a good consistency in frequency distribution. The total wave energy difference rate of the two energy density curves is 27.68%. The total wave energy of the SBF7-1A GPS wave buoy is much smaller than the total wave energy of the DWR-MKIII Waverider buoy. This is mainly due to the fact that the three raw movement velocity data sequences of the SBF7-1A GPS wave buoy have some unusual movement velocity values, such as null velocity data segments with a value of 0.

**Figure 5:**
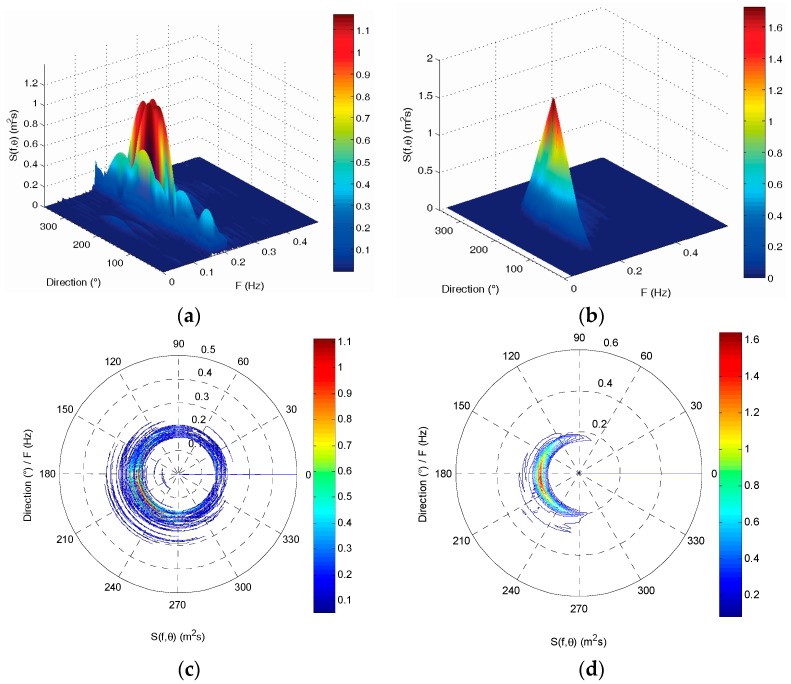
Comparison of directional wave spectrum between the SBF7-1A GPS wave buoy and the DWR-MKIII Waverider buoy before correction at 03:00 (UTC) on 2 July 2013: (**a**,**c**) directional wave spectrum of the SBF7-1A GPS wave buoy; (**b**,**d**) directional wave spectrum of the DWR-MKIII Waverider buoy.

The directional wave spectrum of the two wave buoys at 03:00 (UTC) on 2 July 2013 are shown in Figure 5. They also do not have a good consistency in frequency distribution and direction distribution. The wave energy density distributions measured by the SBF7-1A GPS wave buoy are more dispersed than the DWR-MKIII Waverider buoy. This affects the identification of wave direction from the directional wave spectrum. Additionally, the total wave energy measured by the two buoys is also very different, and the difference rate is also 27.68%.

## 4. Processing with a Neural-Network Algorithm and a Gaussian Low-Pass Filter

For the problem of energy reduction and energy distribution dispersion in wave spectrum, this paper proposes two improvement measures. In the algorithm flow of measuring ocean waves with satellite GPS signals, a neural network and a Gaussian low-pass filter are introduced.

### 4.1. Nonlinear Autoregressive Neural Network

**Figure 6:**
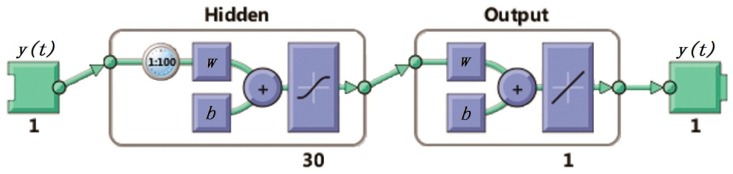
Nonlinear autoregressive neural network.

At present, neural networks have been used in various fields of scientific research. This paper designs a nonlinear autoregressive neural network model for processing raw movement velocity data sequences of the SBF7-1A GPS wave buoy, as shown in Figure 6 and Equation (13).

Nonlinear autoregressive neural networks can be trained to predict a time series based on previous data points. The wave period is mostly between 2 and 25 s. In this paper, 50 s are selected as the prediction period of the nonlinear autoregressive neural network, which is basically twice the wave period. The SBF7-1A GPS wave buoy collects the movement velocity data twice per second according to the satellite GPS signals. There are 100 movement velocity data points within a prediction period of the neural network. In a movement velocity data sequence, missing data points, i.e., data gaps due to that some data have been identified as being unusual, are filled by predicted values based on the nonlinear autoregressive neural network calculation. The nonlinear autoregressive neural network uses 100 consecutive data points before a data gap to determine the predicted value to fill the gap. Through many experiments, it is found that a neural network with 30 hidden layers can achieve the best prediction results.

(13) $$y\left(N\right)=w_{1}y\left(N-1\right)+w_{2}y\left(N-2\right)+w_{3}y\left(N-3\right)+w_{4}y\left(N-4\right)+......+w_{100}y\left(N-100\right)$$

**Figure 7:**
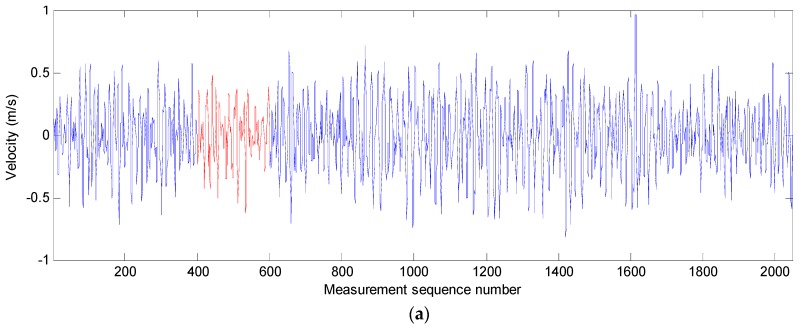

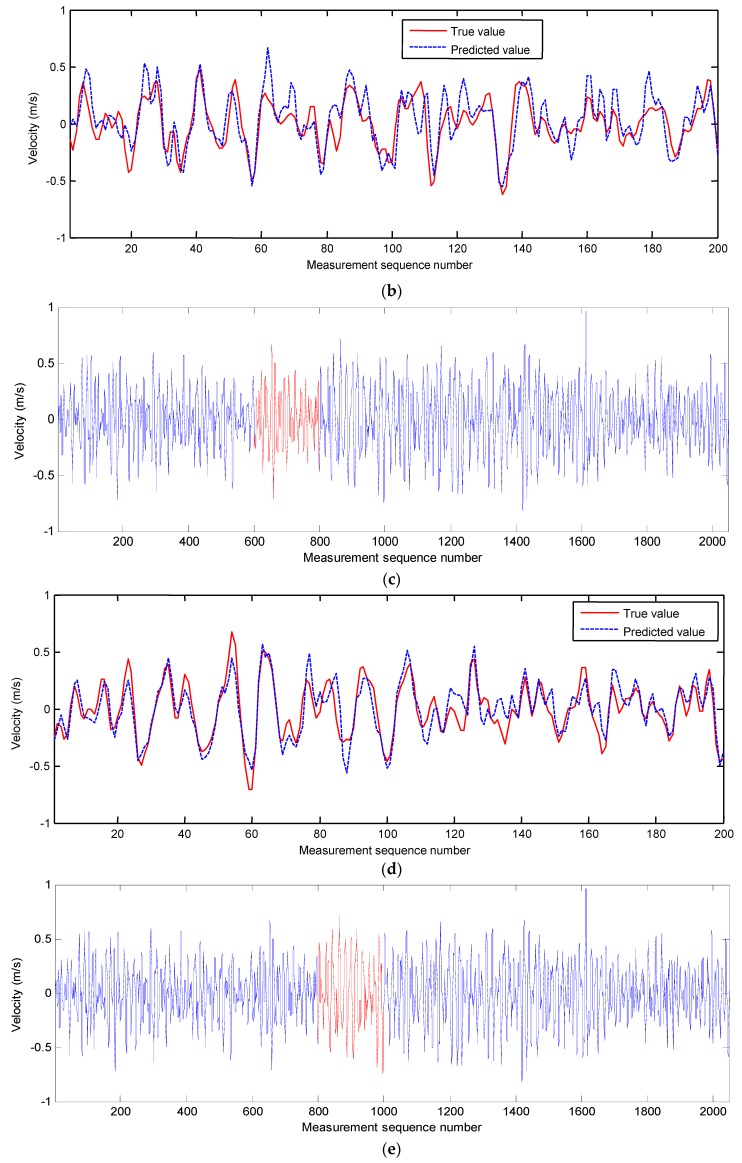

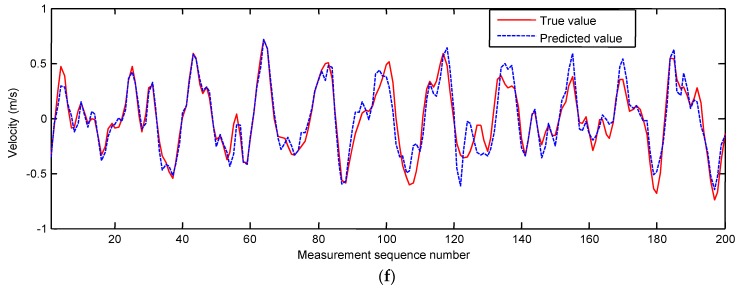
Test cases for the predicting effect of the nonlinear autoregressive neural network; (**a**,**b**) test case 1; (**c**,**d**) test case 2; (**e**,**f**) test case 3.

In order to verify the correction effect of the neural network approach, an artificial gap is used in the movement-velocity data sequence, as shown in Figure 7a,c,e. In a real velocity data sequence of the SBF7-1A GPS wave buoy, the blue data are the measured, and the red data are assumed to be the lost data. The lost data are predicted using the measured data before it according to the neural-network approach. The comparison of true data and predicted data is shown in Figure 7b,d,f and Table 1. It can be seen from the comparison that the more real data before the predicted data, the better the prediction effect. In the three test cases, the correlation coefficients between predicted data and real data are all greater than 0.75, and the errors of velocity data points are all less than 0.5 m/s. Most predicted velocity data points have a small error, and only few data points have a slightly larger error. Their average differences are all less than 0.04 m/s despite the maximum difference value being 0.47 m/s.

Its specific work flow is as follows.

(1) Picking out unusual movement velocity values

From the raw movement velocity data sequence, find unusual velocity values and mark them.

A method has been found to mark some violently rising velocity data from the velocity data sequence. In a velocity data sequence, three consecutive velocity data points are sequentially selected from the beginning of the data sequence to the end of the data sequence. First, the absolute values of the three velocity data points are taken to obtain three absolute values. Then, the three absolute values are averaged to obtain an average value. The absolute value of each velocity data is compared to this average value. When the absolute value of the velocity data is greater than three times the average value, it is considered that the velocity data represent a violent-rising data value and need to be marked as unusual data. 

A method has also been found to mark some data segments with a data value of 0 from the velocity data sequence. In a velocity data sequence, three consecutive velocity data are sequentially selected from the beginning of the data sequence to the end of the data sequence. When the three velocity data values are all 0, the three velocity data values are considered to be unusual data, and the three velocity data values need to be marked at the same time.

(2) Forward training the neural network

In a raw velocity data sequence, all data segments with more than 100 consecutive usual velocity data are selected as samples to train the nonlinear autoregressive neural network.

(3) Forward correcting the raw movement velocity data sequence

In the velocity data sequence, the unusual data mark is sequentially searched from the beginning of the sequence to the end of the sequence. If there are 100 consecutive usual data points before unusual data, the 100 usual data are input into the nonlinear autoregressive neural network to predict a velocity value. This unusual data point is replaced by the predicted velocity value, and is also marked as a usual data in the data sequence.

(4) Reverse training the neural network

After the above operations, sometimes there are still some unusual data in the movement velocity data sequence. Therefore, the velocity data sequence needs to be corrected in reverse direction. The velocity data sequence is arranged in reverse time order. In the reverse velocity data sequence, all data segments with more than 100 consecutive usual velocity data are selected as samples to retrain the nonlinear autoregressive neural network. 

(5) Reverse correcting the raw movement velocity data sequence

In the reverse velocity data sequence, some unusual velocity data are corrected to follow a similar operation to Step 3.

(6) Rearranging the reverse movement velocity data sequence

The reverse velocity data sequence is rearranged into a forward velocity data sequence in time order.

After all the above operations, the unusual data are all corrected in the movement velocity data sequence.

### 4.2. Gaussian Low-Pass Filter

**Figure 8:**
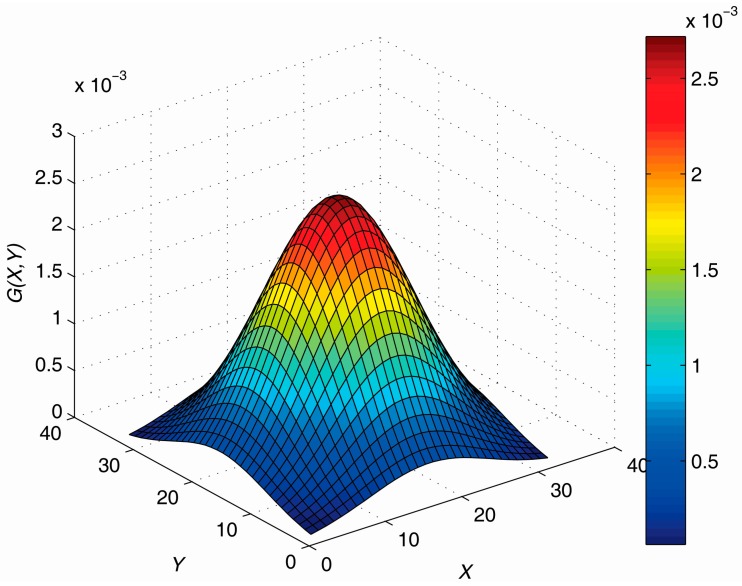
Gaussian low-pass filter.

This paper designs a Gaussian low-pass filter for processing the raw directional wave spectrum of the SBF7-1A GPS wave buoy, as shown in Figure 8 and Equation (14). The Gaussian low-pass filter can concentrate the distribution of wave energy and make the main wave direction more accurate. Through many experiments, it is found that a Gaussian low-pass filter with a frequency space size of 32, direction space size of 32, and standard deviation sigma of 8 can achieve the best smoothing effect.

(14) $$G\left(X,Y\right)=e^{-\frac{\left(X^{2}+Y^{2}\right)}{2\sigma^{2}}}$$

## 5. In Situ Comparison Test and Results with a Neural-Network Algorithm and a Gaussian Low-Pass Filter

**Figure 9:**
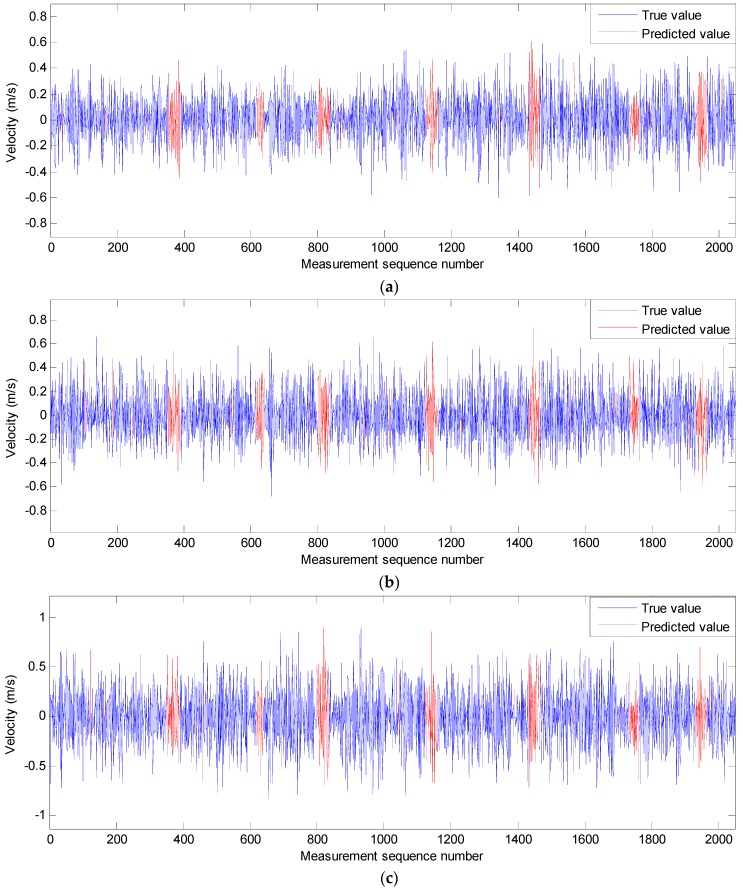
Movement velocity data sequences of the SBF7-1A GPS wave buoy in three directions after correction at 03:00 (UTC) on 2 July 2013: (**a**) movement velocity data sequence in the east-west direction; (**b**) movement velocity data sequence in the north–south direction; (**c**) movement velocity data sequence in the vertical direction.

After being corrected with a nonlinear autoregressive neural network, the three movement velocity data sequences are shown in Figure 9. There are no unusual velocity data in these sequences.

By using a neural network algorithm and a Gaussian low-pass filter, the wave frequency spectrum and the directional wave spectrum are calculated from the three corrected movement velocity data sequences. The wave height, wave period, wave direction, wave frequency spectrum, and directional wave spectrum measured by the SBF7-1A GPS wave buoy and the DWR-MKIII Waverider buoy in the same time period are compared. Correlation analysis is performed on the measurement results of each physical quantity from the two devices.

**Figure 10:**
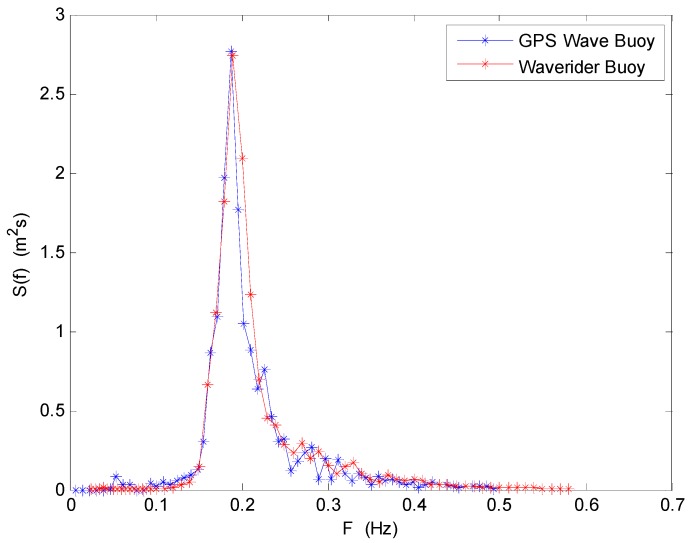
Comparison of wave frequency spectrum between the SBF7-1A GPS wave buoy and the DWR-MKIII Waverider buoy after correction at 03:00 (UTC) on 2 July 2013.

The wave frequency spectrum of the two wave buoys at 03:00 (UTC) on 2 July 2013 are shown in Figure 10. They have very good consistency in frequency distribution. The wave energy density distributions measured by the two buoys are all concentrated in the frequency range from 0.15 to 0.25 Hz, and the energy density values all reach an extreme value at a frequency of about 0.2 Hz. The total wave energy difference rate of the two energy density curves is only 4.86%.

**Figure 11:**
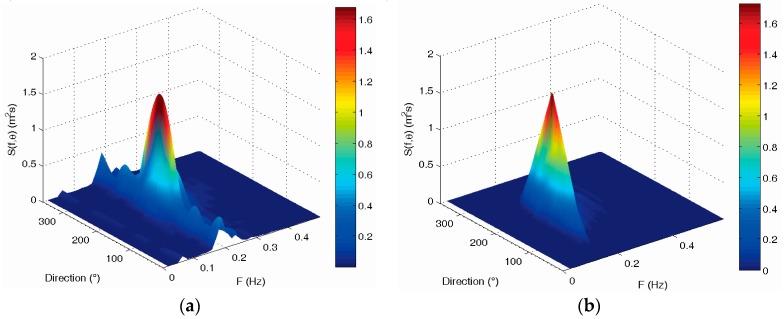

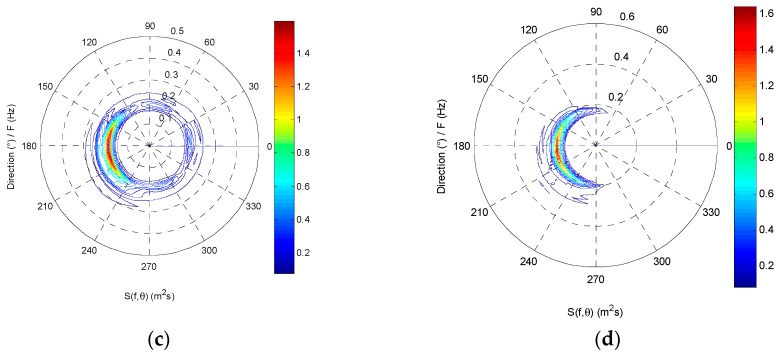
Comparison of directional wave spectrum between the SBF7-1A GPS wave buoy and the DWR-MKIII Waverider buoy after correction at 03:00 (UTC) on 2 July 2013: (**a**,**c**) directional wave spectrum of the SBF7-1A GPS wave buoy; (**b**,**d**) directional wave spectrum of the DWR-MKIII Waverider buoy.

The directional wave spectrum of the two wave buoys at 03:00 (UTC) on 2 July 2013 are shown in Figure 11. They also have very good consistency in frequency distribution and direction distribution. The wave energy density distributions measured by the two buoys are all concentrated in the direction range from 120 to 240 degrees. The total wave energy measured by the two buoys is very consistent, and the difference rate is also only 4.86%.

**Figure 12:**
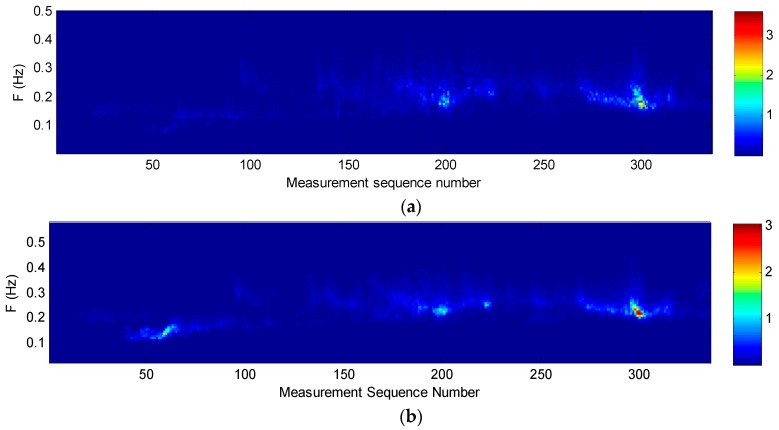
Comparison of time-sequence images of wave frequency spectrum between the SBF7-1A GPS wave buoy and the DWR-MKIII Waverider buoy after correction from 19 June to 3 July 2013: (**a**) SBF7-1A GPS wave buoy; (**b**) DWR-MKIII Waverider buoy.

The wave frequency spectrums of the two buoys during the field test are respectively arranged to form time-sequence images as the measurement time (Figure 12). It can be seen that the wave energy density distributions of the two buoys are also very consistent in the time and frequency domain.

**Figure 13:**
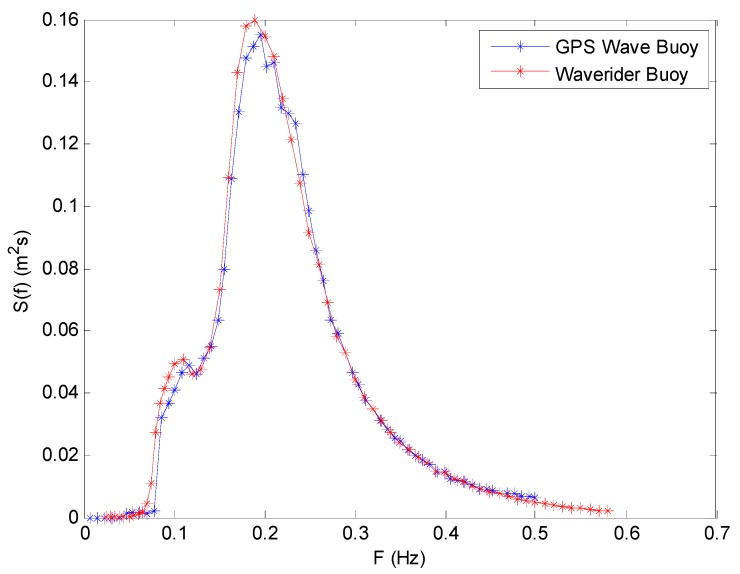
Comparison of average wave frequency spectrum between the SBF7-1A GPS wave buoy and the DWR-MKIII Waverider buoy after correction from 19 June to 3 July 2013.

All the wave frequency spectrums of the two buoys are first summed and then averaged to obtain an average wave frequency spectrum, respectively. Their comparison is shown in Figure 13. Their energy density distribution curves are also very consistent. The total wave energy difference rate of the two energy density distribution curves is only 5.61%.

**Figure 14:**
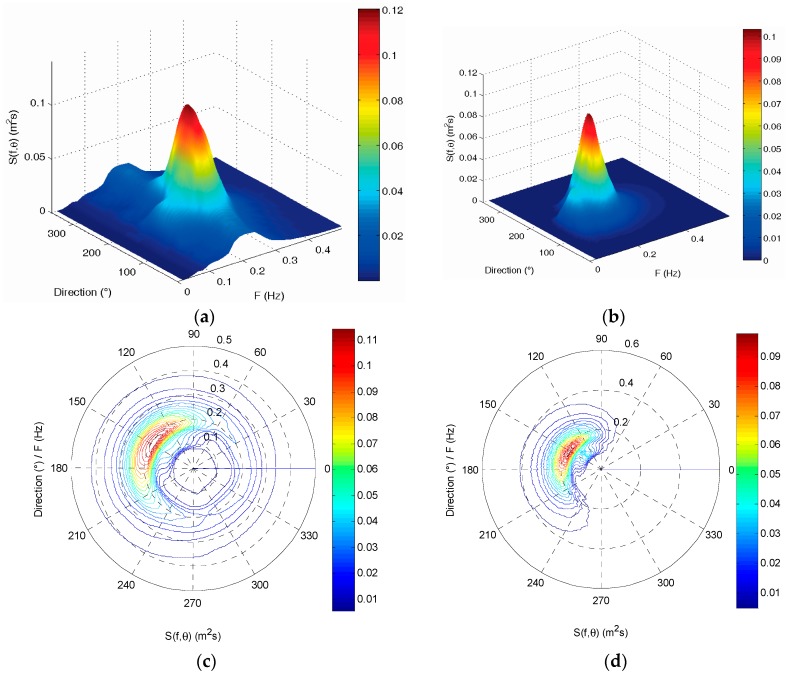
Comparison of average directional wave spectrum between the SBF7-1A GPS wave buoy and the DWR-MKIII Waverider buoy after correction from 19 June to 3 July 2013: (**a**,**c**) average directional wave spectrum of the SBF7-1A GPS wave buoy; (**b**,**d**) average directional wave spectrum of the DWR-MKIII Waverider buoy.

All the directional wave spectrums of the two buoys are first summed and then averaged to obtain an average directional wave spectrum, respectively. Their comparison is shown in Figure 14. Their energy density distribution maps are also very consistent in frequency distribution and direction distribution. The wave energy distributions of the two buoys are all concentrated in the same wave direction range from 90 to 210 degrees.

**Figure 15:**
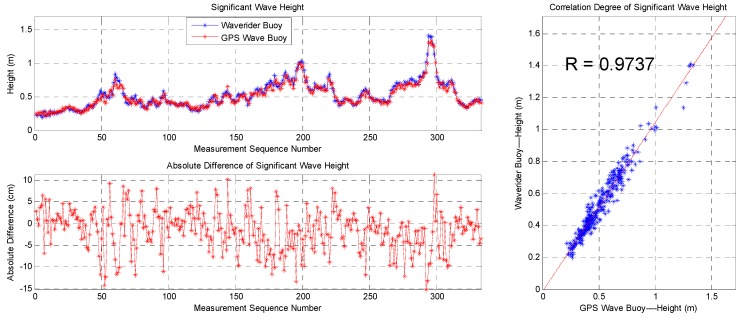
Comparison of significant wave heights between the SBF7-1A GPS wave buoy and the DWR-MKIII Waverider buoy after correction.

The data sequence of the significant wave height and statistics of the data from the two buoys are shown in Figure 15 and Table 2, respectively. The correlation coefficient of the significant wave heights using the two devices is 0.9737, their average difference is –1.58 cm, the root-mean-square difference is 5.02 cm, and the standard deviation is 4.76 cm, indicating good consistency. Most differences are below 10 cm despite the maximum value being 15.14 cm.

**Figure 16:**
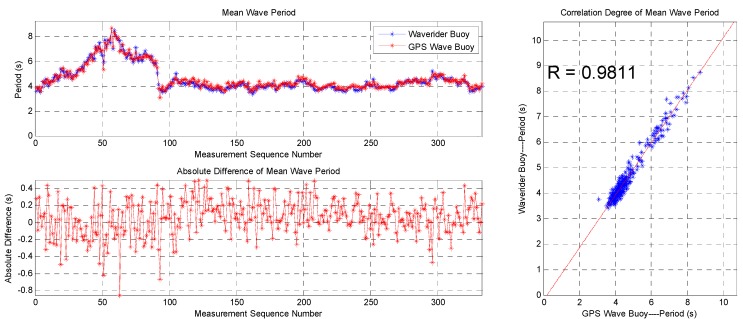
Comparison of mean wave periods between the SBF7-1A GPS wave buoy and the DWR-MKIII Waverider buoy after correction.

The data sequence of the mean wave period and statistics of the data from the two buoys are shown in Figure 16 and Table 2, respectively. The correlation coefficient is 0.9811, the average difference is 0.06 s, the root-mean-square difference is 0.22 s, and the standard deviation is 0.21 s, indicating good consistency. Most of the absolute differences of the mean wave period are below 0.5 s despite the maximum value being 0.85 s.

**Figure 17:**
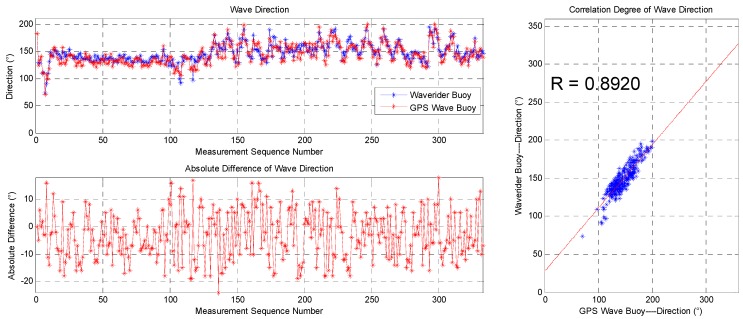
Comparison of wave directions between the SBF7-1A GPS wave buoy and the DWR-MKIII Waverider buoy after correction.

The data sequence of the wave direction and statistics of the data from the two devices are shown in Figure 17 and Table 2, respectively. The correlation coefficient is 0.8920, the average difference is −3.19 degrees, the root-mean-square difference is 9.27 degrees, and the standard deviation is 8.70 degrees, indicating good consistency. Most of the absolute differences of the wave direction are below 10 degrees despite the maximum value being 24.00 degrees.

## 6. Deep Sea Measurement Test and Results from the SBF7-1A GPS Wave Buoy

**Figure 18:**
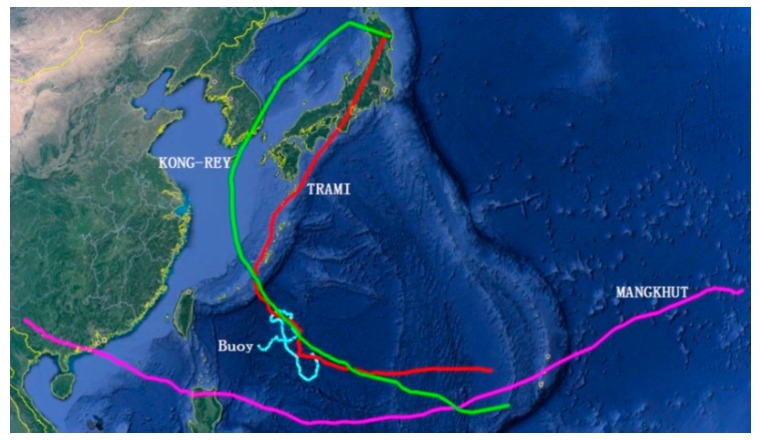
Moving paths from the SBF7-1A GPS wave buoy, Typhoons MANGKHUT, TRAMI, and KONG-REY.

**Figure 19:**
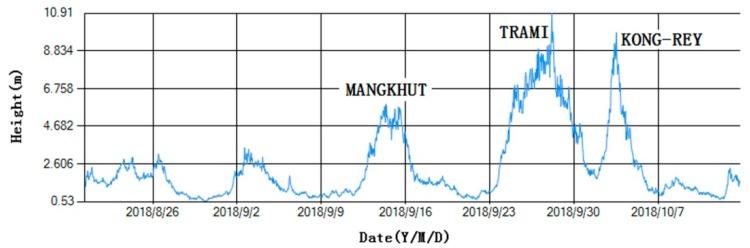
Data curve of significant wave heights from the SBF7-1A GPS wave buoy during three typhoons.

In order to test reliability under severe sea states, the SBF7-1A GPS wave buoy was placed in the Western Pacificon on 18 August 2018. It still worked very stably after experiencing Typhoons MANGKHUT, TRAMI, and KONG-REY, as shown in Figure 18 and Figure 19 and Table 3. In particular, the buoy measured a significant wave height of 10.91 m at 20:00 (UTC) on 27 September 2018. At this time, the distance between the buoy and the typhoon eye of TRAMI was about 103 km.

At the same time, compared to results derived from observations with remote sensing satellites, the differences of significant wave height from the two devices were also very small, as shown in Table 4.

Currently, the buoy is still working properly. The measuring accuracy and measuring reliability of the SBF7-1A GPS wave buoy under severe sea states have been verified again.

## 7. Discussion

This paper describes a method for measuring ocean waves using satellite GPS signals, which is different from the traditional method of measuring ocean waves using acceleration. At the same time, this paper also streamlines the algorithmic flow of GPS measurement wave and proposes two improvement measures. A nonlinear autoregressive neural network is introduced to correct unusual movement velocity data. A Gaussian low-pass filter is introduced to smooth the directional wave spectrum. It can be seen that the wave measurement algorithm and the two improvement measures proposed in this paper are feasible from the field test results of the SBF7-1A GPS wave buoy and the DWR-MKIII Waverider buoy. The correlation coefficients are greater than 0.97, 0.98, and 0.89 in significant wave height, mean wave period, and wave direction, respectively. The differences are mostly within 10 cm, 0.5 s, and 10 degrees in significant wave height, mean wave period, and wave direction, respectively. The wave energy density distributions are also very consistent in energy amplitude, frequency space, and direction space. 

However, it is necessary to further improve the preprocessing method in the raw movement velocity data sequences of the GPS wave buoy, because the GPS antenna is often covered by sea water, which affects receiving satellite GPS signals and solving the movement velocity. As such, unusual movement velocity data often appear. The smaller the number of continuous unusual velocity data, the better the correction effect of the neural network. When there are too many continuous unusual velocity data, the correction effect of the neural network is reduced. Therefore, the next step is to combine the neural network algorithm model and other methods to further improve the correction effect in the case of a large number of consecutive unusual movement velocity data. 

At the same time, the sea surface is rough, and some GPS signals from multiple satellites and multiple directions will be reflected by the sea surface to the GPS antenna. The multipath GPS reflection signals can further increase the error of the measurement velocity. We have tested a brand of GPS receiver that can output position information in the house. In theory, it should not output position information because the GPS satellite signals are blocked in the house. Our analysis shows that the main reason for this phenomenon is that the GPS receiver receives the GPS signals reflected from the glass inside the house. We have proved through sea trials that the brand GPS receiver has a very large error in measuring waves. Finally, we chose another brand of GPS receiver that can greatly eliminate the effects of multipath GPS reflection signals and improve the accuracy of measuring waves.

Next, in order to further eliminate the effects of multipath GPS reflection signals, we intend to take the following measures. First, the GPS wave buoy selects a brand of GPS antenna that is resistant to multipath effects. Second, a blocking plate is placed around the GPS antenna to block some low-angle multipath signals. Finally, the original satellite signals data are filtered to further eliminate multipath effects [25,26,27].

## 8. Conclusions

Through the sea trials of the SBF7-1A GPS wave buoy and the DWR-MKIII Waverider buoy, it can be seen that the two devices are in good consistency in terms of wave height, wave period, wave direction, wave frequency spectrum, and directional wave spectrum even under severe sea states. The discrepancies are within a reasonable range. The maximum difference of the significant wave height is less than 0.2 m plus 10% of the true wave height value. The maximum discrepancy of the mean wave period is less than 1 s. The maximum discrepancy of the wave direction is also less than 30 degrees. This paper shows the measurement accuracy and reliability of the SBF7-1A GPS wave buoy under severe sea states. Therefore, the feasibility of wave measurement with satellite GPS signals has been verified. At the same time, it further shows that the SBF7-1A GPS wave buoy developed by the National Ocean Technology Center, China has reached the advanced technological level of similar products in the world.

At present, the Chinese Beidou satellite system is under construction. Moreover, it has started to provide navigation and positioning services, and its measurement accuracy is gradually increasing. Therefore, future research work should simultaneously use satellite GNSS signals from the Beidou, GPS, Glonass, and Galileo systems to further improve the accuracy, reliability, safety, and redundancy of wave measurements.

## Figures and Tables

**Table 1:** Comparison of results between the true velocity data sequence and the predicted velocity data sequence.

	Correlation Coefficient	Average Difference (m/s)	Maximum Difference (m/s)	Minimum Difference (m/s)	Root-Mean-Square Difference (m/s)	Standard Deviation (m/s)
Test case 1	0.7754	−0.03	0.44	−0.47	0.16	0.16
Test case 2	0.8712	−0.009	0.30	−0.39	0.12	0.12
Test case 3	0.9361	−0.003	0.28	−0.34	0.11	0.11

**Table 2:** Comparison of results between the SBF7-1A GPS wave buoy and the DWR-MKIII Waverider buoy after correction.

	Correlation Coefficient	Average Difference	Maximum Difference	Minimum Difference	Root-Mean-Square Difference	Standard Deviation
Significant wave height (cm)	0.9737	–1.58	11.35	–15.14	5.02	4.76
Mean wave period (s)	0.9811	0.06	0.50	–0.85	0.22	0.21
Wave direction (°)	0.8920	–3.19	18.00	–24.00	9.27	8.70

**Table 3:** Maximums of significant wave height from the SBF7-1A GPS wave buoy in three typhoons.

Typhoon	Date (YMD)	Time (UTC)	Distance (Buoy and Typhoon Eye)	Significant Wave Height of Buoy
MANGKHUT	2018-09-14	01:00	about 630 km	5.90 m
TRAMI	2018-09-27	20:00	about 103 km	10.91 m
KONG-REY	2018-10-03	05:00	about 126 km	9.85 m

**Table 4:** Comparison of significant wave heights between the SBF7-1A GPS wave buoy and remote sensing satellites.

Date (YMD)	Time (UTC)	Satellite (Saral or Jason-3)	Significant Wave Height of Buoy	Significant Wave Height of Satellite	Difference
20180828	01:00	Jason-3	0.85 m	1.05 m	–0.20 m
20180830	00:00	Jason-3	0.56 m	0.71 m	–0.15 m
20180902	21:00	Saral	2.81 m	2.92 m	–0.11 m
20180915	21:00	Saral	3.66 m	3.80 m	–0.14 m
